# Transparent, High Glass-Transition Temperature, Shape Memory Hybrid Polyimides Based on Polyhedral Oligomeric Silsesquioxane

**DOI:** 10.3390/polym11061058

**Published:** 2019-06-18

**Authors:** Zhongxu Lan, Xueli Chen, Xiao Zhang, Chongyu Zhu, Yanlei Yu, Jia Wei

**Affiliations:** Department of Materials Science & State Key Laboratory of Molecular Engineering of Polymers, Fudan University, Shanghai 200433, China; 16110300006@fudan.edu.cn (Z.L.); 18210300029@fudan.edu.cn (X.C.); 16110300018@fudan.edu.cn (X.Z.); cy_zhu@fudan.edu.cn (C.Z.); ylyu@fudan.edu.cn (Y.Y.)

**Keywords:** polyimide, POSS, optical transparency, thermal stability, shape memory effect

## Abstract

Optically transparent polyimides with excellent thermal stability and shape memory effect have potential applications in optoelectronic devices and aerospace industries. A series of optically transparent shape memory polyimide hybrid films are synthesized from 2,2-bis(3,4-dicarboxyphenyl)hexafluoropropane dianhydride (6FDA) and 2,2′-bis-(trifluoromethyl)biphenyl-4,4′-diamine (TFMB) with various polyhedral oligomeric silsesquioxane (POSS) contents and then subjected to thermal imidization. The hybrid films show good optical transparency (>80% at 400 nm and >95% at 500 nm) with cutoff wavelengths ranging from 318 to 336 nm. Following the incorporation of the inorganic POSS structure, the hybrid films exhibit excellent thermal stability with glass transition temperature (*T*_g_) ranging from 351 to 372 °C. The hybrid films possess the highest *T*_g_ compared with the previously-reported shape memory polymers. These findings show that POSS is successfully utilized to develop transparent polyimides with excellent thermal stability and shape memory effect.

## 1. Introduction

Polyimide (PI) is widely used in the microelectronics and aerospace industries because of its excellent thermal stability, chemical resistance and suitability for advanced manufacturing [1,2,3]. Optically transparent PIs with high thermal resistance have drawn great attention due to their extensive applications as transparent flexible substrates of optoelectronic devices, such as image displays, organic photovoltaics, printing circuit boards, and touch panels [4]. High temperature-resistant transparent PIs with shape memory effect, which involve conversion into a temporary shape and restoration to the original shape under external stimuli, have potential in advanced adaptive optoelectronic and deployable aerospace structural applications [2,5,6,7,8,9,10,11,12,13,14,15,16,17,18]. However, the trade-off between good transparency and thermal stability of PI films remains challenging due to the contradiction between the two properties in many instances [4].

A favorable strategy for achieving colorless and transparent PI is to use weak electron-donating diamines or weak electron-accepting dianhydrides to suppress charge transfer (CT) interactions [19,20,21]. Improving the optical properties of PI has been attempted by introducing highly electronegative fluorine groups [22,23], alicyclic moieties [24,25], asymmetric segments [26,27], and bulky substituents [28,29,30]. Unfortunately, these methods diminish the thermal stability in many cases. For example, replacing aromatic dianhydride PMDA (1,2,4,5-benzenetetracarboxylic anhydride) with fluorinated dianhydride, such as 2,2-bis(3,4-dicarboxyphenyl)hexafluoropropane dianhydride (6FDA), decreased *T*_g_ by up to 50 °C [31]. Methodologies increasing the thermal stability of PI, such as by introducing highly-conjugated substituents and rigid aromatic groups, usually sacrifice optical transparency [1]. Compared with 6FDA, aromatic dianhydride PMDA endowed PI with better thermal stability, while the transmittance decreased by about 50% at 450 nm simultaneously [32]. By contrast, incorporating inorganic materials improved the thermal stability and minimally affected the optical transparency of the PI [33,34]. These inorganic materials mainly include organoclay and polyhedral oligomeric silsesquioxane (POSS).

Owing to the nanosized, rigid and stereo structure of cage-like silsesquioxane, POSS molecules are considered as the smallest nanoreinforcements of organic polymers to improve the polymers’ properties [35,36]. The introduction of POSS to PI makes the resulted hybrid polymers exhibit advantages in low dielectric constants [37,38], flame retardance [39], atomic oxygen resistance [40,41], especially good thermal stability, and thermo-dimensional stability [42,43,44,45]. Improvement of the thermal stability is mainly attributed to the rigidity increasement of the polymer chain due to the presence of POSS molecules [46,47]. Besides, the inclusion of proper POSS molecules in PI possibly does not give rise to deterioration of optical properties [42]. Octafunctional POSS with eight polymerizable sites is an ideal candidate to design a hybrid polyimide network, providing a solution to trade-off between good transparency and thermal stability. The copolymerization of POSS with PI will leads to dramatic improvements of thermal properties, and the molecular level dispersion of POSS in the polymer matrix is very important to maintain the optical properties of resulting nanocomposites. Furthermore, the POSS netpoints also endow the polymer with shape memory effect.

Generally, shape memory PI is divided into thermoplastic and thermoset PI by physical crosslinking and chemical crosslinking respectively [8,9,10,12,17]. Thanks to the covalently crosslinked three-dimensional networks, the property of the thermoset shape memory PI is prior to that of the thermoplastic shape memory PI in many aspects, such as better mechanical properties, good chemical resistance, excellent thermal stability, and better shape memory performances [48]. Hence, thermoset shape memory PI is expected to be a promising high-performance material in the field of deployable space structures, high temperature sensors and actuators [8].

In this study, we obtain a series of thermoset PI-POSS hybrid films with good transparency, excellent thermal stability and shape memory effect by copolymerizing of octa(aminophenyl)-silsesquioxane (OAPS) and fluorinated monomers (6FDA/TFMB). Fluorine-contained diamine and dianhydride are used as monomers to render the PI films good transparency. OAPS is introduced as a co-monomer to improve thermal stability and provide the films with shape memory effect. To the best of our knowledge, the resulted films possess the highest *T*_g_ compared with the reported shape memory polymers (SMPs).

## 2. Experimental

### 2.1. Materials

2,2-bis(3,4-dicarboxyphenyl)hexafluoropropane dianhydride (6FDA) and 2,2′-bis-(trifluoro-methyl)biphenyl-4,4′-diamine (TFMB) were purchased from CHINATECH (TIANJIN) CHEMICAL CO., LTD (Tianjin, China). Anhydrous grade dimethylacetamide (DMAc) and tetrahydrofuran (THF) were purchased from Adamas (Shanghai, China). OAPS was synthesized following the procedure reported elsewhere [49,50]. Other reagents were used as received. The synthetic route and characterizations of OAPS are shown in Figures S1–S4.

### 2.2. Preparation of PI-POSS Hybrid Films

**Scheme 1:**
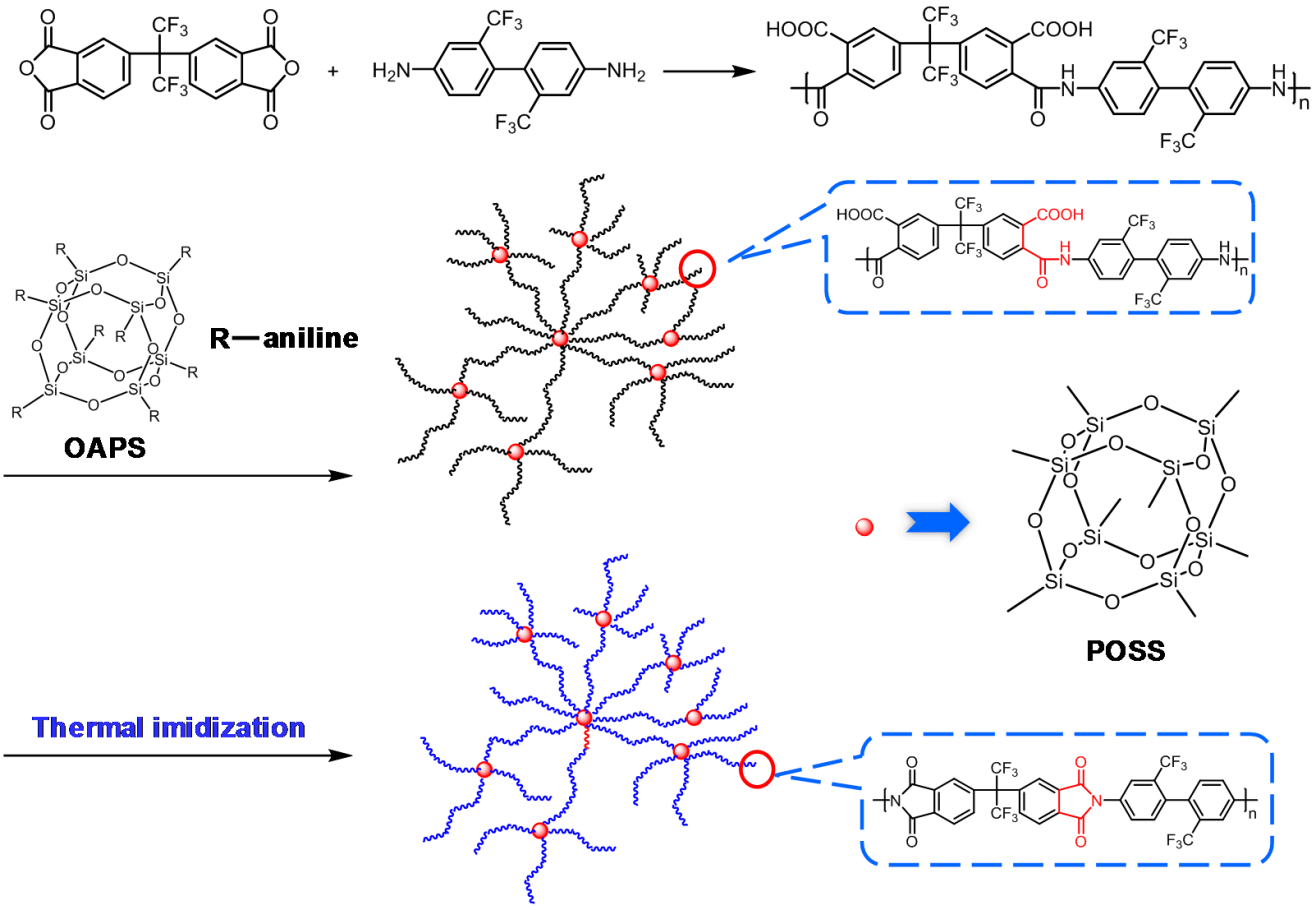
Synthesis of PI-POSS hybrids.

PI-POSS hybrids were prepared via a conventional two-step method (Scheme 1) and the compositions of the PI-POSS hybrid films are listed in Table S1. The mole ratio of NH_2_ in the OAPS to the total NH_2_ was 0, 1/20, 2/20, 3/20, and 4/20, and the feed weight percent of OAPS was consequently varied to include 0, 1.9, 3.8, 5.7, and 7.6 wt%, corresponding to PI-1, PI-2, PI-3, PI-4 and PI-5 respectively. The solid content in the reaction mixture was kept at 15 wt%. For example, PI-2 was synthesized through the following procedures: a 25-mL three-necked flask equipped with a nitrogen inlet and a magnetic stirrer was charged with TFMB (3.04 g, 9.5 mmol) and 20 mL DMAc under nitrogen atmosphere. An accurate quantity of 6FDA (4.44 g, 10 mmol) in 20 mL DMAc was added after TFMB dissolved completely. The mixture was stirred at room temperature for 6 h, and then a solution of OAPS (0.144 g, 0.125 mmol) in 6 mL THF was added to the system. The mixture was continuously stirred for another 2 h to obtain the PAA (polyamic acid) solution. The PAA solution was then cast on smooth glass substrates by spin-coating technique and thermally imidized at 60, 80, 100, 200 and 300 °C for 1 h respectively. After imidization, the transparent PI-POSS hybrid films were immersed in deionized water to facilitate removal from the glass substrates and dried at 100 °C in a blast oven.

### 2.3. Characterization

Fourier transform infrared (FT-IR) spectra were recorded with a VERTEX 70v spectrometer (Karlsruhe, Germany) ranging from 4000 to 400 cm^−1^ at a resolution of 4 cm^−1^ using transmission mode with KBr pellet. ^1^H NMR spectra were collected using a Bruker AVANCE III spectrometer (400 MHz, Karlsruhe, Germany) in acetone-*d*_6_ or dimethylsulfoxide-*d*_6_. ^29^Si NMR spectra were recorded on a Bruker 400WB AVANCE III spectrometer (Karlsruhe, Germany). Dynamic mechanical analysis (DMA) was carried out with a TA instrument Q800 (New Castle, DE, USA) using tension mode in air at a heating rate of 3 °C/min ranging from 50 to 400 °C with a frequency of 1 Hz and an amplitude of 15 µm, the samples were cut into 30 mm (L) × 3 mm (W) × 15 µm (T). Differential scanning calorimetry (DSC) was carried out with a TA instrument Q2000 at a heating rate of 10 °C/min ranging from 50 to 400 °C in 40 mL/min nitrogen flow with about 2 mg samples, and the second heating cycle was used to determine the glass transition temperature. The coefficient of thermal expansion (CTE) values of PI-POSS hybrid films (20 mm (L) × 5 mm (W) × 15 µm (T)) in the glassy state were measured as an average from 50–200 °C by TMA at a heating rate of 5 °C/min in 40 mL/min nitrogen flow and the second heating cycle was used to determine the CTE values. Thermogravimetric analysis (TGA) was performed on a TA instrument Q500 (New Castle, DE, USA) at a heating rate of 20 °C/min ranging from 50 to 900 °C in 40 mL/min nitrogen flow with about 5 mg samples. The mechanical properties of the PI-POSS films (25 mm (L) × 5 mm (W) × 15 µm (T)) were tested on an Instron universal tester 5567 at ambient temperature at a crosshead speed of 6 mm/min. For each sample, three samples were tested and the average value was used. Ultra violet-visible (UV-vis) spectra of the PI-POSS hybrid films were recorded on a Lambda 650 UV/VIS spectrometer (Waltham, MA, USA) from 200 to 800 nm at a resolution of 2 nm.

The PI-POSS hybrid film was cut into the shape of a flower to show the shape memory effect. The “petals” of the “flower” were bent on the surface of the hot stage (30 °C higher than *T*_g_), and the temporary shape was fixed by removing the “flower” from the hot stage to room temperature. Then the “flower” recovered to its original shape when it was placed back onto the hot stage. The shape recovery process was repeated several cycles and recorded by using a digital camera.

The stretchable shape memory effect of the PI-POSS hybrid film was measured by TMA in 40 mL/min nitrogen flow with a preload of 0.003 N. The procedure includes the following steps: (1) heating the sample to *T*_g_ + 30 °C at 20 °C/min, (2) applying a force to elongate the sample, (3) reducing the temperature to *T*_g_ − 170 °C at 20 °C/min, (4) force removal and holding the sample for another 5 min, (5) reheating to *T*_g_ + 30 °C at 10 °C/min and holding for 30 min to trigger recovery.

Shape recovery (*R*_r_) and shape fixity (*R*_f_) are two important factors in evaluating the shape memory effect. The *R*_r_ value represents the ability of the material to recover to its permanent shape, and the *R*_f_ value shows the ability of the material to hold the external force to maintain the deformation. These variables are calculated using Equations (1) and (2), respectively.

(1) $$R_{r}\left(N\right)=\frac{\varepsilon_{m}\left(N\right)-\varepsilon_{p}\left(N\right)}{\varepsilon_{m}\left(N\right)-\varepsilon_{p}\left(N-1\right)}\times 100\%$$

*ε*_m_, *ε*_p_ and N represent strain after stretching step (before cooling), strain after recovery and cycle number.

(2) $$R_{f}\left(N\right)=\frac{\varepsilon_{u}\left(N\right)}{\varepsilon_{m}\left(N\right)}\times 100\%$$

*ε*_u_ represents strain in the fixed temporary shape.

## 3. Results and Discussion

### 3.1. Thermal Behavior

**Figure 1:**
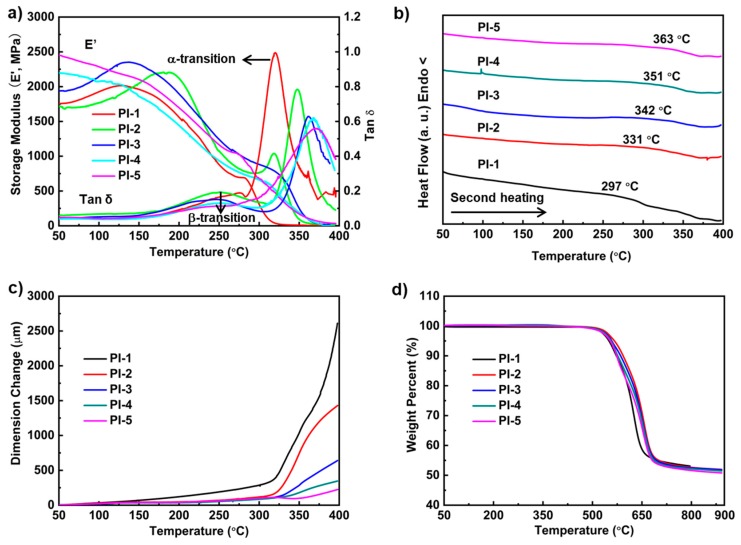
(**a**) DMA, (**b**) DSC, (**c**) TMA, and (**d**) TGA curves of pure PI and PI-POSS hybrid films.

As a very successful colorless aromatic PI, 6FDA/TFMB PI exhibits high transparency in the visible region, whereas the thermal properties are not good enough (*T*_g_ ~ 300 °C and CTE ~ 150 ppm/°C) to resist the high processing temperature during the fabrication of flexible display [3]. Based on this case, octafunctional POSS was combined with 6FDA/TFMB to obtain hybrid PI to improve the thermal properties. The thermal stability was investigated for all the obtained PI-POSS and pure PI in an effort to understand the influence of POSS content. The glass transition temperature of the PI-POSS hybrid film with different OAPS concentrations was evaluated by DMA and DSC (Figure 1a,b and Table 1). The DMA test indicated that the hybrid films (PI-2 to PI-5) possessed increased *T*_g_ and higher storage modulus (E’) compared with the pure PI film (PI-1). PI-5 showed *T*_g_ of 372 °C (50 °C higher) and glassy state storage modulus of 2.45 GPa at 50 °C (40% higher) compared with that of PI-1. All films possessed similar β-transition temperatures due to the movements of the side chain (CF_3_), whereas the α-transition caused by the segmental motion of the backbone obviously shifted to higher temperature with increasing OAPS content. The storage modulus improved considerably with increasing OAPS content. The *T*_g_ of the films obtained from DSC also showed considerable enhancement from 297 to 362 °C as OAPS content increased. Notably, PI-2 exhibited increased *T*_g_ (30 °C higher than that of PI-1) with only 1.9 wt% OAPS content, suggesting that *T*_g_ was improved obviously even at a very low OAPS concentration. The effective enhancement of *T*_g_ was attributed to the rigid cage structure of POSS and the crosslinking network provided by the polymerizable OAPS. The presence of POSS restricted the motion of the polymer chain and improved *T*_g_ in agreement with the literature [51].

The thermal dimensional stability of PI films was represented by the coefficient of thermal expansion (CTE). The CTE values showed a sharp decline with the introduction of OAPS (Figure 1c and Table 1). When the OAPS loading reached 5.7 wt%, the CTE value decreased by approximately 70% compared with that of pure PI (141 ppm/°C) and reached 42 ppm/°C. This improvement was also attributed to the crosslinking network provided by the rigid POSS.

The thermogravimetric analysis of the PI-POSS hybrid film is shown in Figure 1d and the results are listed in Table 1. As shown in Table 1, little improvement in both *T*_d_ and *T*_d_^5^ was observed with increasing OAPS loading. The *T*_d_ of PI-5 was 546 °C at 7.6 wt% OAPS loading, increasing by 26 °C compared with that of PI-1. The char yield of all films was over 50% at 800 °C. Thermal studies clearly indicated that incorporation of POSS cage in the polymer can drastically modify the thermal properties, allowing for the tailoring of the *T*_g_, CTE and decomposition temperature by changing the POSS content.

### 3.2. Optical Properties

**Figure 2:**
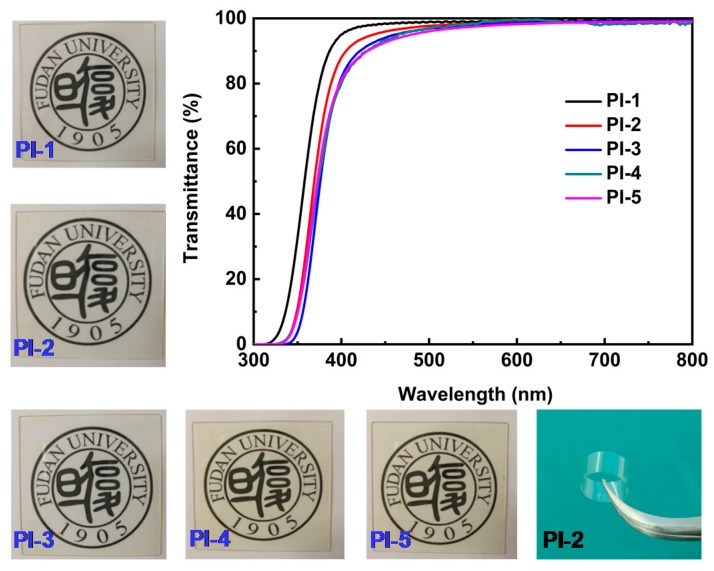
Photographs and transmittance of pure PI and PI-POSS hybrid films with thickness about 15 µm.

**Figure 3:**
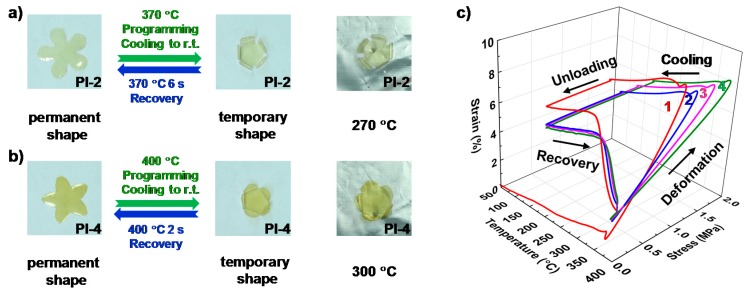
(**a**) Shape memory of flower-shaped PI-2 with thickness about 50 μm. The permanent shape was placed on a hot stage at 370 °C and bent with a certain angle, followed by cooling down to room temperature to fix the temporary shape. Then it recovered to permanent shape in 6 s when it was placed back onto the 370 °C hot stage. It can keep the temporary shape on the 270 °C hot stage. (**b**) Shape memory of flower-shaped PI-4 with thickness about 30 μm. The permanent shape was placed on a hot stage at 400 °C and bent with a certain angle, followed by cooling down to room temperature to fix the temporary shape. Then it recovered to permanent shape in 2 s when it was placed back into the 400 °C hot stage. It can keep the temporary shape on the 300 °C hot stage. (**c**) Shape memory cycle in TMA of PI-4. An external force was applied at 400 °C to deform a specimen to a certain strain. Then the specimen was cooled down (20 °C/min) to 200 °C, the stress released, and the specimen equilibrated for 5 min and then reheated (10 °C/min) to 400 °C to trigger recovery.

Figure 2 presents photographs and UV-vis spectra of the PI-POSS hybrid films as well as pure PI film with thickness about 15 µm. All films had good optical transmittance (*T*_400_ > 80% and *T*_500_ > 95%) in the visible region and a low cutoff wavelength (*λ*_0_) ranging from 310 to 336 nm. PI-2 with 1.9 wt% OAPS loading maintained 88% transmittance at 400 nm and remained colorless. As OAPS loading increased from 3.8 to 9.6 wt%, *T*_400_ of the hybrid films ranged from 80 to 82%. The slight decrease of transmittance was due to trace colored impurity contained in the OAPS monomer. Besides, the thickness of the film also affected the optical transmittance. For instance, PI-2 with a thickness of about 15 µm in Figure 2 was almost colorless, while it turned into pale yellow in Figure 3a with thickness increasing to 50 µm.

The reasons why the PI-POSS hybrid films possessed good transmittance in the visible region are summarized as follows. First and foremost, the existence of the CF_3_ groups loosened the PI chain stacking which was advantageous for reducing inter-molecular CT interactions, resulting in a decrease of film coloration and increase of transmittance [4,52]. Second, the introduction of POSS molecules as a rigid component did not bring a large sacrifice around the 400-nm region because no conjugated aromatic or heterocyclic groups were involved. Third, the way to incorporate POSS as crosslinkers by polymerization effectively avoided aggregation of POSS which always takes place in hybrid polymers containing nonfunctional POSS due to the strong POSS–POSS interactions. The molecular level dispersion of nanofiller in the polymer matrix was very important to maintain the optical properties of resulting nanocomposites.

Here, the overall performance of the PI-POSS films, such as *T*_g_, *T*_d_^5^, *λ*_0_, and *T*_400_, was compared with the available system reported recently. A novel alicyclic dianhydride, 2*R*,5*R*,7*S*,10*S*-naphthanetetracarboxylic dianhydride (HNTDA), was prepared to fabricate colorless polyimides. Among the resultant polyimides, HNTDA/APB (APB ~ 1,4-bis (4-amino-phenoxy) benzene) possessed a higher *T*_g_ (357 °C) and good optical properties (*λ*_0_ ~ 325 nm and *T*_400_ ~ 80%), while the *T*_d_^5^ was only 482 °C and lower than those of conventional aromatic polyimides [53]. An alkyl chain-bridged amino-polyhedral oligomeric silsesquioxane (ABA-POSS) was obtained and used to fabricate PI-organosilicate composite materials. The transmittance of the PI films was over 90% between 400 nm and 700 nm, while the *T*_g_ values were between 250–280 °C [42]. In this work, the PI-POSS films displayed high thermal stability (*T*_g_ ~ 351–372 °C and *T*_d_^5^ ~ 552–566 °C) due to the fluorinated aromatic structure and crosslinking network provided by OAPS, good optical properties (*λ*_0_ ~ 318–336 nm and *T*_400_ ~ 80–88%) attributed to weak CT interactions, and homogeneous dispersion of POSS molecules. The films also possessed good mechanical properties due to the rigid structure of the polyimide backbone and crosslinking network (Table S1).

### 3.3. Shape Memory Effect

Shape memory polymers (SMPs) with high triggering temperature have wide potential applications in harsh conditions, such as high temperature sensors and actuators, deployable space structures, and shape-morphing structures [54,55,56,57]. Although shape memory PIs with high *T*_g_ ranging from 299 to 322 °C can be obtained, the color remained very deep because of the large absorption in the visible region [8]. Recently, a kind of shape memory PI with transmittance higher than 81% at 450–800 nm was also reported by the same research group [9]. In order to endow the film with good transparency, the *T*_g_ of the PI was sacrificed and reduced to 171 °C, which is still higher than other transparent SMPs reported.

In the present work, we inferred that the polyimide chain acted as a reversible phase, the chemical crosslinking provided by OAPS acted as a permanent phase, and the existence of a crosslinking network would endow the hybrid films with a shape memory effect. Here we took PI-2 (*T*_g_ ~ 351 °C) and PI-4 (*T*_g_ ~ 369 °C) as examples to study the shape memory effect. The flower-shaped PI-2 and PI-4 films were bent with a certain angle on a hot stage at 370 and 400 °C respectively, followed by cooling down to room temperature to fix the temporary shape (Videos S1 and S2). To observe the shape recovery process, the “flowers” with the temporary shape were placed back onto the hot stage at 370 and 400 °C (Videos S3 and S4). The typical images of the shape memory process of PI-2 and PI-4 are shown in Figure 3a,b. It took just 6 s and 2 s for the flower-shaped PI-2 and PI-4 films to recover to permanent shape from temporary shape, indicating the hybrid films had a short recovery time. To exhibit good shape recovery ability, the PI-4 film was cut into a stripe and bent to an acute angle at 400 °C and cooled down to room temperature to fix the temporary shape, then it was placed back onto the 400 °C hot stage and recovered to permanent shape in 5 s (Video S5).

It is well known that the triggering temperature of SMPs can be a crystallization temperature, glass transition temperature, or the temperature at which physical crosslinking reverses [58,59,60]. In this work, the triggering temperature was the glass transition temperature. The high *T*_g_ of the hybrid films indicated that the materials may have little to no creep at room temperature and long periods of storage without performance deterioration [12]. Moreover, the high *T*_g_ of the hybrid films avoided inadvertent triggering for the application in different environments and temperatures. In order to observe the high triggering temperature clearly, the flower-shaped PI-2 and PI-4 films with temporary shape were heated from 270 to 370 °C and 300 to 400 °C at 50 °C/min respectively. We found they could maintain the temporary shape at 270 and 300 °C (Videos S6 and S7) stably. With the increase of temperature, the temporary shape began to recover to permanent shape when the temperature was close to *T*_g_ of the two films (Videos S8 and S9).

The shape memory process of PI-4 was repeated many times, and the film appeared to recover to its original shape every time with no observed damage in Video S10. Video S11 demonstrated that PI-1 with no crosslinking did not possess the shape memory effect in contrast with PI-2 and PI-4, which clarified the important role of POSS in the shape memory effect.

Shape recovery (*R*_r_) and shape fixity (*R*_f_) were characterized with consecutive shape memory cycles determined by TMA (Figure 3c). Typically, a rectangular specimen of PI-4 was heated to 400 °C (30 °C higher than *T*_g_). The specimen was stretched at 400 °C and then cooled down to 200 °C (170 °C lower than *T*_g_) to fix the temporary shape. The force was then removed and the specimen was reheated to 400 °C to trigger recovery. *R*_f_ values of the first, second, third, and fourth cycles were 80.2, 82.5, 81.9, and 82.8%, whereas *R*_r_ values were 66.6, 97.5, 99.1, and 99.5%, respectively. The difference of *R*_r_ among the first and the following cycles was attributed to the residual strain in the film processing history and the plastic deformation generated within the first cycle [7]. The hybrid film displayed quite a good reproducible shape memory effect after the first cycle.

## 4. Conclusions

A series of organic–inorganic polyimides with POSS as crosslinkers were successfully synthesized by the copolymerization of TFMB, 6FDA and OAPS. The electron-withdrawing CF_3_ groups loosen the chain stacking, reducing the inter-molecular CT interactions, and result in a decrease of film coloration and increased transmittance (*T*_400_ > 80% and *T*_500_ > 95%). The incorporation of rigid polymerizable OAPS molecules renders the hybrid films with excellent thermal stability and shape memory effect with the highest *T*_g_ ranging from 351 to 372 °C and short recovery time (2–6 s) compared with the reported SMPs. The high *T*_g_ also provides the hybrid films with resistance to stress relaxation in the temporary shape. In summary, this work has introduced a method to obtain optically transparent polyimide films with excellent thermal stability and shape memory effect, which expands the scope of applications in optoelectronic engineering and aerospace industries.

## Figures and Tables

**Table 1:** The thermal and optical properties of pure PI and PI-POSS hybrid films.

Sample	OAPS (wt%) ^a^	*T*_g_ (°C) ^b^	*T*_d_ (°C) ^c^	*T*_d_^5^ (°C) ^d^	CTE (ppm/K) ^e^	λ_0_ (nm) ^f^	*T*_400_ (%) ^g^	*T*_500_ (%) ^h^
PI-1	0	320	520	550	141	310	95	99
PI-2	1.9	351	528	552	57	326	88	98
PI-3	3.8	363	529	555	43	336	82	97
PI-4	5.7	369	537	557	42	318	80	97
PI-5	7.6	372	546	566	53	326	81	96

^a^ The theoretical weight percent of OAPS in the hybrid films. ^b^ The glass transition temperature was measured by DMA. ^c^ The onset decomposition temperature was measured by TGA in N_2_. ^d^ The 5% weight decomposition temperature was measured by TGA in N_2_. ^e^ The coefficient of thermal expansion was measured by TMA. ^f^ Cutoff wavelength. ^g^ Optical transmittance at 400 nm. ^h^ Optical transmittance at 500 nm.

## Supplementary Materials

The following are available online at https://zenodo.org/record/3248730#.XQi3DsQRVPY, Figure S1: Synthesis of OAPS; Figure S2: FT-IR spectra of ONPS and OAPS; Figure S3: (a) ^1^H NMR (400 MHz, acetone-*d*_6_, δ): 8.7 (t, 1 H), 8.4–8.0 (m, 2 H), 7.8 (m, 2 H). (b) ^1^H NMR (400 MHz, DMSO-*d*_6_, δ): 7.3–6.2 (t, 2 H), 5.2–4.5 (s, 1 H); Figure S4: ^29^Si NMR spectra of ONPS and OAPS; Table S1: The components and mechanical properties of pure PI and PI-POSS hybrid films. Video S1: Fabrication of temporary shape of flower-shaped PI-2 film; Video S2: Fabrication of temporary shape of flower-shaped PI-4 film; Video S3: Shape recovery of flower-shaped PI-2 film; Video S4: Shape recovery of flower-shaped PI-4 film; Video S5: Shape memory of PI-4 stripe; Video S6: Temporary shape of flower-shaped PI-2 film on 270 °C hot stage; Video S7: Temporary shape of flower-shaped PI-4 film on 300 °C hot stage; Video S8: Shape recovery of flower-shaped PI-2 film through heating from 270 to 370 °C; Video S9: Shape recovery of flower-shaped PI-4 film through heating from 300 to 400 °C; Video S10: Shape memory cycles of PI-4; Video S11: Shape memory text of PI-1.

## References

[1] Ji D.Y., Hu W.P., Fuchs H. (2019). Recent Progress in Aromatic Polyimide Dielectrics for Organic Electronic Devices and Circuits. Adv. Mater..

[2] Gouzman I., Crossman E., Verker R., Atar N., Bolker A., Eliaz N. (2019). Advances in Polyimide-Based Materials for Space Applications. Adv. Mater..

[3] Blanco I., Cicala G., Ognibene G., Rapisarda M., Recca A. (2018). Thermal properties of polyetherimide/polycarbonate blends for advanced applications. Polym. Degrad. Stab..

[4] Tapaswi P.K., Ha C.S. (2019). Recent Trends on Transparent Colorless Polyimides with Balanced Thermal and Optical Properties: Design and Synthesis. Macromol. Chem. Phys..

[5] Gao H., Huang L.N., Lan X., Liu L.W., Liu Y.J., Leng J.S. The research of vacuum thermal cycling resistant transparent shape memory polyimide and its application in space flexible electronics. Proceedings of the SPIE Smart Structures and Materials + Nondestructive Evaluation and Health Monitoring.

[6] Gao H., Lan X., Liu L.W., Xiao X.L., Liu Y.J., Leng J.S. (2017). Study on performances of colorless and transparent shape memory polyimide film in space thermal cycling, atomic oxygen and ultraviolet irradiation environments. Smart Mater. Struct..

[7] Xiao X.L., Kong D.Y., Qiu X.Y., Zhang W.B., Liu Y.J., Zhang S., Zhang F.H., Hu Y., Leng J.S. (2015). Shape memory polymers with high and low temperature resistant properties. Sci. Rep..

[8] Xiao X.L., Kong D.Y., Qiu X.Y., Zhang W.B., Zhang F.H., Liu L.W., Liu Y.J., Zhang S., Hu Y., Leng J.S. (2015). Shape-Memory Polymers with Adjustable High Glass Transition Temperatures. Macromolecules.

[9] Xiao X.L., Qiu X.Y., Kong D.Y., Zhang W.B., Liu Y.J., Leng J.S. (2016). Optically transparent high temperature shape memory polymers. Soft Matter.

[10] Gao H., Li J.R., Xie F., Liu Y.J., Leng J.S. (2018). A novel low colored and transparent shape memory copolyimide and its durability in space thermal cycling environments. Polymer.

[11] Kong D.Y., Xiao X.L. (2016). High Cycle-life Shape Memory Polymer at High Temperature. Sci. Rep..

[12] Koerner H., Strong R.J., Smith M.L., Wang D.H., Tan L.S., Lee K.M., White T.J., Vaia R.A. (2013). Polymer design for high temperature shape memory: Low crosslink density polyimides. Polymer.

[13] Yang Z.H., Wang Q.H., Wang T.M. (2016). Tunable Triple-Shape Memory Binary Mixtures with High Transition Temperature and Robust Mechanical Properties. Macromol. Chem. Phys..

[14] Yang Z.H., Chen Y., Wang Q.H., Wang T.M. (2016). High performance multiple-shape memory behaviors of Poly(benzoxazole-co-imide)s. Polymer.

[15] Yang Z.H., Wang Q.H., Bai Y.K., Wang T.M. (2015). AO-resistant shape memory polyimide/silica composites with excellent thermal stability and mechanical properties. RSC Adv..

[16] Bai Y.K., Mao L., Liu Y.J. (2016). High temperature shape memory polyimide ionomer. J. Appl. Polym. Sci..

[17] Wang Q.H., Bai Y.K., Chen Y., Ju J.P., Zheng F., Wang T.M. (2015). High performance shape memory polyimides based on π–π interactions. J. Mater. Chem. A.

[18] Yang Z.H., Song F.Z., Wang Q.H., Wang T.M. (2016). Shape Memory Induced Structural Evolution of High Performance Copolyimides. J. Polym. Sci. Part A Polym. Chem..

[19] Wakita J., Ando S. (2009). Characterization of Electronic Transitions in Polyimide Films Based on Spectral Variations Induced by Hydrostatic Pressures up to 400 MPa. J. Phys. Chem. B.

[20] Sato Y., Yoshida M., Ando S. (2006). Optical Properties of Rod-like Fluorinated Polyimides and Model Compounds Derived from Diamines having High Electron-donating Properties. J. Photopolym. Sci. Technol..

[21] Takizawa K., Wakita J., Azami S., Ando S. (2011). Relationship between Molecular Aggregation Structures and Optical Properties of Polyimide Films Analyzed by Synchrotron Wide-Angle X-ray Diffraction, Infrared Absorption, and UV/Visible Absorption Spectroscopy at Very High Pressure. Macromolecules.

[22] Zhai L., Yang S.Y., Fan L. (2012). Preparation and characterization of highly transparent and colorless semi-aromatic polyimide films derived from alicyclic dianhydride and aromatic diamines. Polymer.

[23] Hasegawa M., Hirano D., Fujii M., Haga M., Takezawa E., Yamaguchi S., Ishikawa A., Kagayama T. (2013). Solution-Processable Colorless Polyimides Derived from Hydrogenated Pyromellitic Dianhydride with Controlled Steric Structure. J. Polym. Sci. Part A Polym. Chem..

[24] Hasegawa M. (2001). Semi-aromatic polyimides with low dielectric constant and low CTE. High Perform. Polym..

[25] Hsiao S.H., Wang H.M., Chen W.J., Lee T.M., Leu C.M. (2011). Synthesis and Properties of Novel Triptycene-Based Polyimides. J. Polym. Sci. Part A Polym. Chem..

[26] Han K.S., You K., Jang W.H., Rhee T.H. (2000). Synthesis and properties of chlorinated polyimides. Macromol. Chem. Phys..

[27] Hsiao S.H., Yang C.P., Chen S.H. (2000). Synthesis and Properties of Ortho-Linked Aromatic Polyimides Based on 1,2-Bis(4-aminophenoxy)-4-tertbutylbenzene. J. Polym. Sci. Part A Polym. Chem..

[28] Tapaswi P.K., Choi M.C., Jeong K.M., Ando S., Ha C.S. (2015). Transparent Aromatic Polyimides Derived from Thiophenyl-Substituted Benzidines with High Refractive Index and Small Birefringence. Macromolecules.

[29] Huang W., Yan D.Y., Lu Q.H. (2001). Synthesis and Characterization of a Highly Soluble Aromatic Polyimide from 4,4′-Methylenebis(2-tertbutylaniline). Macromol. Rapid Commun..

[30] Liaw D.J., Chang F.C., Leung M.K., Chou M.Y., Muellen K. (2005). High Thermal Stability and Rigid Rod of Novel Organosoluble Polyimides and Polyamides Based on Bulky and Noncoplanar Naphthalene-Biphenyldiamine. Macromolecules.

[31] Yeo H., Goh M., Ku B.C., You N.H. (2015). Synthesis and characterization of highly-fluorinated colorless polyimides derived from 4,4′-((perfluoro-[1,1′-biphenyl]-4,4′-diyl)bis(oxy))bis(2,6-dimethylaniline) and aromatic dianhydrides. Polymer.

[32] Guan Y., Wang D.M., Song G.L., Dang G.D., Chen C.H., Zhou H.W., Zhao X.G. (2014). Novel soluble polyimides derived from 2,2′-bis[4 -(5-amino-2-pyridinoxy)phenyl]hexafluoropropane: Preparation, characterization, and optical, dielectric properties. Polymer.

[33] Choi C.H., Sohn B.H., Chang J.H. (2013). Colorless and transparent polyimide nanocomposites: Comparison of the properties of homo- and co-polymers. J. Ind. Eng. Chem..

[34] Moon K.H., Chae B., Kim K.S., Lee S.W., Jung Y.M. (2019). Preparation and Characterization of Transparent Polyimide–Silica Composite Films Using Polyimide with Carboxylic Acid Groups. Polymers.

[35] Mohamed M.G., Kuo S.W. (2019). Functional Polyimide/Polyhedral Oligomeric Silsesquioxane Nanocomposites. Polymers.

[36] Blanco I. (2018). The Rediscovery of POSS: A Molecule Rather than a Filler. Polymers.

[37] Lee Y.J., Huang J.M., Kuo S.W., Lu J.S., Chang F.C. (2005). Polyimide and polyhedral oligomeric silsesquioxane nanocomposites for low-dielectric applications. Polymer.

[38] Wu S.M., Hayakawa T., Kikuchi R., Grunzinger S.J., Kakimoto M., Oikawa H. (2007). Synthesis and Characterization of Semiaromatic Polyimides Containing POSS in Main Chain Derived from Double-Decker-Shaped Silsesquioxane. Macromolecules.

[39] Devaraju S., Vengatesan M.R., Selvi M., Kumar A.A., Alagar M. (2012). Synthesis and characterization of bisphenol-A ether diamine-based polyimide POSS nanocomposites for low K dielectric and flame-retardant applications. High Perform. Polym..

[40] Minton T.K., Wright M.E., Tomczak S.J., Marquez S.A., Shen L.H., Brunsvold A.L., Cooper R., Zhang J.M., Vij V., Guenthner A.J. (2012). Atomic Oxygen Effects on POSS Polyimides in Low Earth Orbit. ACS Appl. Mater. Interfaces.

[41] Li X.B., Al-Ostaz A., Jaradat M., Rahmani F., Nouranian S., Rushing G., Manasrah A., Alkhateb H., Finckenor M., Lichtenhan J. (2017). Substantially enhanced durability of polyhedral oligomeric silsequioxane-polyimide nanocomposites against atomic oxygen erosion. J. Eur. Polym. J..

[42] Jung Y., Byun S., Park S., Lee H. (2014). Polyimide−Organosilicate Hybrids with Improved Thermal and Optical Properties. ACS Appl. Mater. Interfaces.

[43] Liao W.H., Yang S.Y., Hsiao S.T., Wang Y.S., Li S.M., Ma C.C.M., Tien H.W., Zeng S.J. (2014). Effect of Octa(aminophenyl) Polyhedral Oligomeric Silsesquioxane Functionalized Graphene Oxide on the Mechanical and Dielectric Properties of Polyimide Composites. ACS Appl. Mater. Interfaces.

[44] Verker R., Grossman E., Eliaz N. (2012). Effect of the POSS–Polyimide nanostructure on its mechanical and electrical properties. Compos. Sci. Technol..

[45] Verker R., Grossman E., Gouzman I., Eliaz N. (2009). TriSilanolPhenyl POSS–polyimide nanocomposites: Structure–properties relationship. Compos. Sci. Technol..

[46] Blanco I., Bottino F.A., Cicala G., Latteri A., Recca A. (2014). Synthesis and Characterization of Differently Substituted Phenyl Hepta Isobutyl-Polyhedral Oligomeric Silsesquioxane/Polystyrene Nanocomposites. Polym. Compos..

[47] Blanco I., Bottino F.A., Cicala G., Cozzo G., Latteri A., Recca A. (2015). Synthesis and thermal characterization of new dumbbell shaped POSS/PS nanocomposites: Influence of the symmetrical structure of the nanoparticles on the dispersion/aggregation in the polymer matrix. Polym. Compos..

[48] Xie F., Huang L.N., Leng J.S., Liu Y.J. (2016). Thermoset shape memory polymers and their composites. J. Intell. Mater. Syst. Struct..

[49] Tamaki R., Tanaka Y., Asuncion M.Z., Choi J.W., Laine R.M. (2001). Octa(aminophenyl)silsesquioxane as a Nanoconstruction Site. J. Am. Chem. Soc..

[50] Ni Y., Zheng S.X., Nie K.M. (2004). Morphology and thermal properties of inorganic–organic hybrids involving epoxy resin and polyhedral oligomeric silsesquioxanes. Polymer.

[51] Blanco I., Abate I., Bottino F.A., Cicala G., Latteri A. (2015). Dumbbell-shaped polyhedral oligomeric silsesquioxanes/polystyrene nanocomposites: The influence of the bridge rigidity on the resistance to thermal degradation. J. Compos. Mater..

[52] Hasegawa M., Horie K. (2001). Photophysics, photochemistry, and optical properties of polyimides. Prog. Polym. Sci..

[53] Hu X.F., Yan J.L., Wang Y.X., Mu H.L., Wang Z.K., Cheng H.Y., Zhao F.Y., Wang Z. (2017). Colorless polyimides derived from 2R,5R,7S,10S-naphthanetetracarboxylic dianhydride. Polym. Chem..

[54] Sokolowski W.M., Tan S.C. (2007). Advanced Self-Deployable Structures for Space Applications. J. Spacecr. Rocket..

[55] Liu Y.J., Du H.Y., Liu L.W., Leng J.S. (2014). Shape memory polymers and their composites in aerospace applications: A review. Smart Mater. Struct..

[56] Paillous A., Pailler C. (1994). Degradation of multiply polymer-matrix composites induced by space environment. Composites.

[57] Wienhold P.D., Persons D.F. (2003). The Development of High-Temperature Composite Solar Array Substrate Panels for the Messenger Spacecraft. SAMPE J..

[58] Behl M., Lendlein A. (2007). Shape-memory polymers. Mater. Today.

[59] Liu C.D., Qin H.H., Mather P.T. (2007). Review of progress in shape-memory polymers. J. Mater. Chem..

[60] Koerner H., Kelley J., George J., Drummy L., Mirau P., Bell N.S., Hsu J.W.P., Vaia R.A. (2009). ZnO Nanorod-Thermoplastic Polyurethane Nanocomposites: Morphology and Shape Memory Performance. Macromolecules.

